# Comparative Assessment of Toxic Metals Bioaccumulation and the Mechanisms of Chromium (Cr) Tolerance and Uptake in *Calotropis procera*

**DOI:** 10.3389/fpls.2020.00883

**Published:** 2020-06-19

**Authors:** Kamal Usman, Hareb Al Jabri, Mohammed H. Abu-Dieyeh, Mohammed H. S. A. Alsafran

**Affiliations:** ^1^Office of Academic Research, Qatar University, Doha, Qatar; ^2^Department of Biological and Environmental Sciences, College of Arts and Sciences, Qatar University, Doha, Qatar

**Keywords:** toxic metals, chromium, plants, *Calotropis procera*, phytoremediation, antioxidant enzymes, superoxide dismutase

## Abstract

Progressive pollution due to toxic metals significantly undermines global environmental sustainability efforts. Chromium (Cr) is one of the most dangerous to human health. The use of plants to rid the environment of such pollutants “phytoremediation” proves to be a promising alternative to the current remediation methods. In the present study, inductively coupled plasma optical emission spectroscopy (ICP-OES) determined Cadmium (Cd), Chromium (Cr), Copper (Cu), Nickel (Ni), and Lead (Pb) concentrations in the soil, and plants (*Atriplex leucoclada*, *Calotropis procera*, *Salsola imbricata*, *Typha augustifolia*, and *Phragmites australis*) root and shoots. Results showed that compared to other studied metals, Cr concentration was the highest in the soil at 111.8 mg/kg, whereas Cd records the least concentration of 0.04 mg/kg. Cr also accumulated in higher concentration in *C. procera* than in the soil and other plants, with up to 188.2 and 68.2 mg/kg concentration in the root and shoot, respectively. In order to understand the mechanism of Cr tolerance and uptake in *C. procera*, germinated seeds were irrigated with 20 mg/kg Cr and control treatment (no Cr applied) for six (6) weeks under greenhouse conditions. Fourier transformed infrared spectroscopy (FTIR) results showed high Cr complexation and binding to *C. procera* tissues via hydroxyl and carboxylic groups. Enzymatic assay reveals increased activities of superoxide dismutase (SOD), catalase (CAT), and glutathione reductase (GR) in Cr treated *C. procera* than in the control. SOD activity increased by up to six (6) folds. Therefore, we conclude that *C. procera* is suitable for the phytoremediation of Cr polluted arid soil. Additionally, regulation of cellular homeostasis via redox signaling is essential to the Cr tolerance and detoxification mechanism.

## Introduction

Environmental pollution due to toxic metals is one of the most pressing environmental issues challenging the global sustainable development agenda. It is especially apparent in countries witnessing rapid industrialization. Metal pollutants gain entry into the air, soil, and water bodies via natural and anthropogenic routes, including domestic and agricultural use, metals mining and smelting activities, and other industrial productions. They are non-biodegradable and persist in the environment for thousands of years (Kushwaha et al., 2018; Usman et al., 2019). Cr, particularly the hexavalent, Cr (VI), is widely used at the industrial level, including stainless steel industries, dye, and leather tanneries (Garg et al., 2007). The United States Environmental Protection (USEPA) listed Cr (VI) as one of the seventeen metals and metalloids that are dangerous to human health. Additionally, Cr (VI) exposure causes severe health hazards to plants and animals exposed (Jobby et al., 2018). A maximum allowable limit of 64 mg/kg Cr is recommended to protect environmental health (Shahid et al., 2017). The use of plants to remove non-essential metals, including Cr, a process termed “phytoremediation,” dates for decades (Nikalje and Suprasanna, 2018). Bioconcentration factor (BCF) and translocation factor (TF) are some of the parameters used in the evaluation of plant metals phytoremediation capacity. BCF measures metals concentration in plant tissues relative to the growth medium, while TF evaluates metals translocation capacity. Several works reviewed in Usman et al. (2018) and Xia et al. (2019), suggests that many plants are capable of removing non-essential metals, including Cr, by phytoremediation However, the desired goal of such technology is still to be achieved, partly due to the poor understanding of the processes and strategies governing toxic metals tolerance and uptake in plants. Therefore, current work is invested in elucidating the mechanisms, particularly at the biochemical and molecular level that underpin not only uptake, but also transport and eventual sequestration in order to optimize promising plants systems for improved phytoremediation of polluted environments (Anjum et al., 2015; Usman et al., 2018; Xia et al., 2019).

Tolerance and bioaccumulation strategies differ between plant species, according to metal type, sources, physical and chemical behavior, and other environmental factors. Among known strategies, phytoextraction seems to be the most efficient (Kumar B. et al., 2017; Xia et al., 2019). Metals translocation is mediated by the xylem and/or phloem cells (Rascio and Navari-Izzo, 2011), and the cationic exchange within plant system alters with the structure of lignin, cellulose, and proteins. Metal stress results in increased accumulation of reactive oxygen species (ROS) (Panda et al., 2007; Kumar R. et al., 2017). The antioxidative system is one mechanism used by plants to deal with the stress through the activities of key enzymes such as superoxide dismutase (SOD), catalase (CAT), and glutathione reductase (GR) (Shahid et al., 2017; Jobby et al., 2018; Usman et al., 2018). SOD is the first line of defense; it sequesters noxious superoxide ions and breaks it into less harmful hydrogen peroxide (H_2_O_2_) and oxygen molecules (O_2_). CAT assists in further detoxification by converting H_2_O_2_ into water (H_2_O) and oxygen molecules (O_2_) (Sidhu et al., 2016; Hasanuzzaman et al., 2018). While GR combats oxidative stress by balancing reduced (GSH) and oxidized glutathione (GSSG).

Plants commonly accumulate Cr in their roots and rarely transfer it to the shoot (Lotfy and Mostafa, 2014). Previous studies reported that the perennial shrub plant, *C. procera* (giant milkweed), may be a useful bioindicator of environmental pollution (Hassan et al., 2015). The plant phytoremediation potential of other toxic metals under different conditions was documented (D’Souza et al., 2010; Al-Yemni et al., 2011; Lottermoser, 2011; Almehdi et al., 2019). However, except for the closely related species, *C. gigantea* (Sangeetha et al., 2019), there is no report on Cr accumulation by *C. procera.* Similarly, other studies established that *C. gigantea* tolerates Cr by exclusion mechanism when the plant leaves collected from polluted environments were analyzed using FTIR. However, there is no report on the binding and complexation mechanism governing Cr uptake with *C. procera.* Further, several studies reviewed in Shahid et al. (2017) and Jobby et al. (2018) linked Cr to reduced nutrient uptake and photosynthetic rate, which negatively affects growth of plants. Consequently, such adverse effects interfere with many morphological, biochemical, and physiological processes. The activation or suppression of antioxidants enzymes in response to metal-induced stress depends on the plant and ROS type (Shahid et al., 2012). Previous work by Rivas et al. (2017) attributes the ability of *C. procera* to thrive in saline environments to its antioxidant system. However, no report on the mechanism of Cr tolerance and uptake in *C. procera* from the antioxidative system perspective. The phytoremediation potential of *C. procera* suggests that it is vital to understand the plant mechanisms of metal stress tolerance. In this regard, the study of plant-metals binding interaction/complexation and specific antioxidative response provides useful insight. Fourier Transformed Infrared Spectroscopy (FTIR) and *in vitro* assay of key antioxidative enzymes are useful in understanding metals binding interaction, complexation, uptake and plants response mechanisms (Wen et al., 2018; Usman et al., 2019).

In the present work, the study area, Mesaieed, is located approximately 40 km from the city center of the state of Qatar. It is home to petrochemical, chemical, steel and aluminum companies with high industrial activities. Therefore, we hypothesized that (i) non-essential metals, including Cr, may contaminate the soil. (ii) The ability of the plant species (*Atriplex leucoclada, Calotropis procera, Salsola imbricata, Typha augustifolia*, and *Phragmites australis*) to grow in the area suggest their non-essential metals (including toxic species) tolerance capacity, and may be useful for phytoremediation purposes. Several non-native plant species were introduced to Qatar. The sampling site adjoins a waterlogged area (similar to a wetland), with the surface runoff of industrial discharges. It is therefore not surprising that *P. australis* and *T. augustifolia*, known to inhabit wetlands, thrive in this environment. Therefore, the study objectives were (i) to assess and compare the concentrations of Cd, Cr, Cu, Ni and Pb in the soil. (ii) Evaluate and compare the metals bioaccumulation in the plants tissues. (iii) Identify plant species with the highest Cr bioaccumulation capacity, and provide insights into the metal binding and uptake mechanism. To achieve the study objectives, samples were first collected and analyzed from Mesaieed. After quantifying metals in the field samples (soil and plant tissues), Cr concentration was found to be among the highest in the soil. In addition, compared to other plants the metal (Cr) recorded higher accumulation in *C. procera* tissues. Therefore, *C. procera* was chosen for a controlled experiment under greenhouse conditions for six (6) weeks to study the plant response to Cr toxicity and gain insight into its tolerance mechanism. For such purpose, and scientific validity, it is important to use young *C. procera* seedlings, more preferably seeds, which are devoid, or with minimal exposure to polluted (non-metal) environment. Therefore, the plant seeds were collected from another area (Al-Gharrafa). Unlike Mesaieed, Al-Gharrafa is a residential area devoid of any industrial activity or known non-essential metals pollution. After 6 weeks of treatment, the harvested plant biomass was analyzed for Cr complexation using FTIR. The activities of SOD, CAT, and GR enzymes were also evaluated.

## Materials and Methods

### The Sampling Site

Mesaieed is an industrial area located south of Doha, in Qatar. It is approximately 40 km from the city center. The area houses major petrochemical, chemical, and steel and aluminum companies. At one end, the sampling site adjoins a waterlogged area (similar to a wetland), with the surface runoff of industrial discharges. Soil and plant species, including *Atriplex leucoclada*, *Calotropis procera, Salsola imbricata*, *Typha augustifolia*, and *Phragmites australis* growing in the area (24°58′03.2″N51°34′26.6″E) were sampled for this study. Total organic carbon was measured with a TOC analyzer equipped with a solid sample module operated at 900°C (Shimadzu 5050A with SSM-5000A; Shimadzu, Kyoto, Japan). The analysis was performed according to ISO 10694 (ISO, 1995). Sample quantity was between 0.5 and 1 g and measurable range from 0.1 to 30 mg OC. Each sample was analyzed in duplicate, and the average reported.

### Measurement of Physical and Chemical Parameters

The basic physicochemical parameters of the study site were determined. Soil pH by digital pH meter (Mettler Toledo FE20 ATC), total soluble salts, and electrical conductivity (EC) (dS m^–1^) by the inductive electromagnetic device (Mettler Toledo S230 SevenCompact) (Rhoades and Corwin, 1981). The total organic carbon (TOC) and total inorganic carbon, hydrogen, and nitrogen (CHN) determined following the method of Walkley (1947) and by using CHNS/O analyzer, Perkin Elmer (2400 CHNS/O Series II System 100V) respectively. All analysis were performed in duplicate, and the averages reported.

### *C. procera* Cr Treatment Under Greenhouse Conditions

Mature *C. procera* fruits were collected from the Al-Gharrafa area (25°19′19.7″N 51°27′14.7″E) of Qatar during 16 November 2019. Seeds were extracted, mixed and pre-treated in concentrated sulfuric acid, and sterilized in 8% sodium hypochlorite. Before Cr (VI) treatment, *C. procera* seeds were germinated in Petri dishes using distilled water and cheesecloths. The young seedlings were used for the experiment (three biological replicates each for treatment and control, and five seedlings per replicate). 20 mg/L Cr in concentration prepared as a treatment by diluting pure ICP-OES Cr standard (1000 mg/L Cr) in a modified Hoagland nutrient solution. While the control contained the modified Hoagland nutrient solution only (i.e., 0 mg/L Cr) (Peralta et al., 2001). Obtained *C. procera* seedlings were then irrigated with 200 mL of 20 mg/L Cr (treatment) or 200 mL Hoagland nutrient solution only (control), every 48 h for 6 weeks. After 6 weeks of treatment and exposure to 120 mg/L Cr, the plant tissues were collected, thoroughly washed in deionized water for Cr bioaccumulation analysis.

### Metals Quantitation Using ICP-OES

Metals quantitation in soil and plants (*A. leucoclada*, *C. procera*, *S. imbricata*, *T. augustifolia*, and *P. australis*) shoot and root was performed as previously described in Usman et al. (2019). Briefly, about 0.5 g (plants) and 0.25 g (soil) samples were digested in nitric acid (HNO_3_) and hydrogen peroxide (H_2_O_2_) or hydrogen fluoride (HF) using a large capacity Environmental Express SC154 HotBlock^®^ digestion system at alternating temperature until solutions were clear. Digested samples were analyzed in Inductively Coupled Plasma Optical Emission Spectrometry (ICP-OES). All samples were in three replicates and metals concentrations measured against Standard Reference Materials (SRM’s), Soil, 2709a, and Apple leaves 1515. SRM’s recovery of all metals ranged from 96–102 and 95.5–99% for Soil and Apples leaves, respectively.

### BCF and TF Computation

The BCF (ratio of metals concentration in plant tissues to metals concentration in the soil) and TF (ratio of metals concentration in the shoot to metals concentration in the root) were evaluated as previously reported in Raj et al. (2020b) and Usman et al. (2019).

### FTIR Analysis

FTIR analysis was performed for Cr treated (20 mg/L Cr) and untreated (0 mg/L Cr) *C. procera* root and shoot from the greenhouse experiment, as previously described in Usman et al. (2019) with slight modifications. About 1 mg of tissue samples in KBr analyzed by FTS-135 (Bio-Rad) spectrometer; spectra data recorded within the 400–4000 Cm^–1^ range. For each sample, 25 scans were processed in three replicates, and infrared spectra data analyzed using origin 9.0 software.

### Enzymes Assay

Enzyme extraction followed the method of Mishra et al. (2006). Briefly, 0.2 g of the fresh tissue sample is homogenized in an ice-cooled mortar using 5 mL 10 mM potassium phosphate buffer (p.H 7.0), 1% (w/v) polyvinylpyrrolidone and 0.1 mM Ethylenediaminetetraacetic acid (EDTA). Centrifugation of homogenate followed under 4°C for 15 min at 15000 g. Enzymatic activities were determined and expressed as Unit/mg of protein. SOD (EC 1.15.1.1) activity measured by determining nitrobluetetrazolium (NBT) photochemical reduction inhibition, as described by Beauchamp and Fridovich (1971). A total of 3 mL assay mixture was used, after 15 min under illumination, and absorbance recorded. While the non-illuminated mixture was used as the control. CAT (EC 1.11.1.6) assay was performed, according to Zhang et al. (2009). A total reaction mixture of 3 mL was prepared, and absorbance recorded at 240 nm for 4 min. While the activity of GR (EC 1.8.1.7), which catalyzes the reduction of glutathione disulfide (GSSG) to the sulfhydryl form glutathione (GSH) was determined following the method of Rao et al. (1996).

### Statistical Analysis

One-way ANOVA) and Pearson’s correlation coefficients (*r*) statistics were performed. Statistical significance was considered at *P* < 0.05. All statistical analysis was performed in three sample replicates.

## Results

### Analysis of Physical and Chemical Parameters

In this section, Table 1 shows the physical and chemical parameters of the study site, Mesaieed. A pH of 7.3 ± 0.2, salinity stood at 2.1 ± 0.3 ppt, while electrical conductivity was 3.89 ± 0.24 mS/m, indicating the soil had a neutral pH and saline nature. Meanwhile, total organic carbon (TOC) was 4.1 ± 0.08%, whereas the total inorganic carbon, nitrogen, and hydrogen stood at 8.8 ± 0.04, 0.06 ± 0.33, and 0.48 ± 0.05%, respectively, revealing the soil to be neutral and saline in nature, suggesting low organic matter and high iron and clay components (Usman et al., 2019). The physicochemical characteristics reflect typical Qatari soil known for its high calcium-magnesium carbonates composition (Peng et al., 2016).

**TABLE 1:** Physical and chemical properties of the soil at Mesaieed (*n* = 5) ± SEM.

**pH**	**EC (mS/m)**	**TOC (%)**	**Inorganic Carbon; C, Nitrogen; N and Hydrogen; H (%)**	**Salinity (ppt)**
7.03 ± 0.2	3.89 ± 0.24	4.1 ± 0.08	C: 8.8 ± 0.4 N: 0.06 ± 0.33 H: 0.48 ± 0.05	2.1 ± 0.3

### Metals Concentration in the Soil and Plants Root and Shoot

**FIGURE 1:**
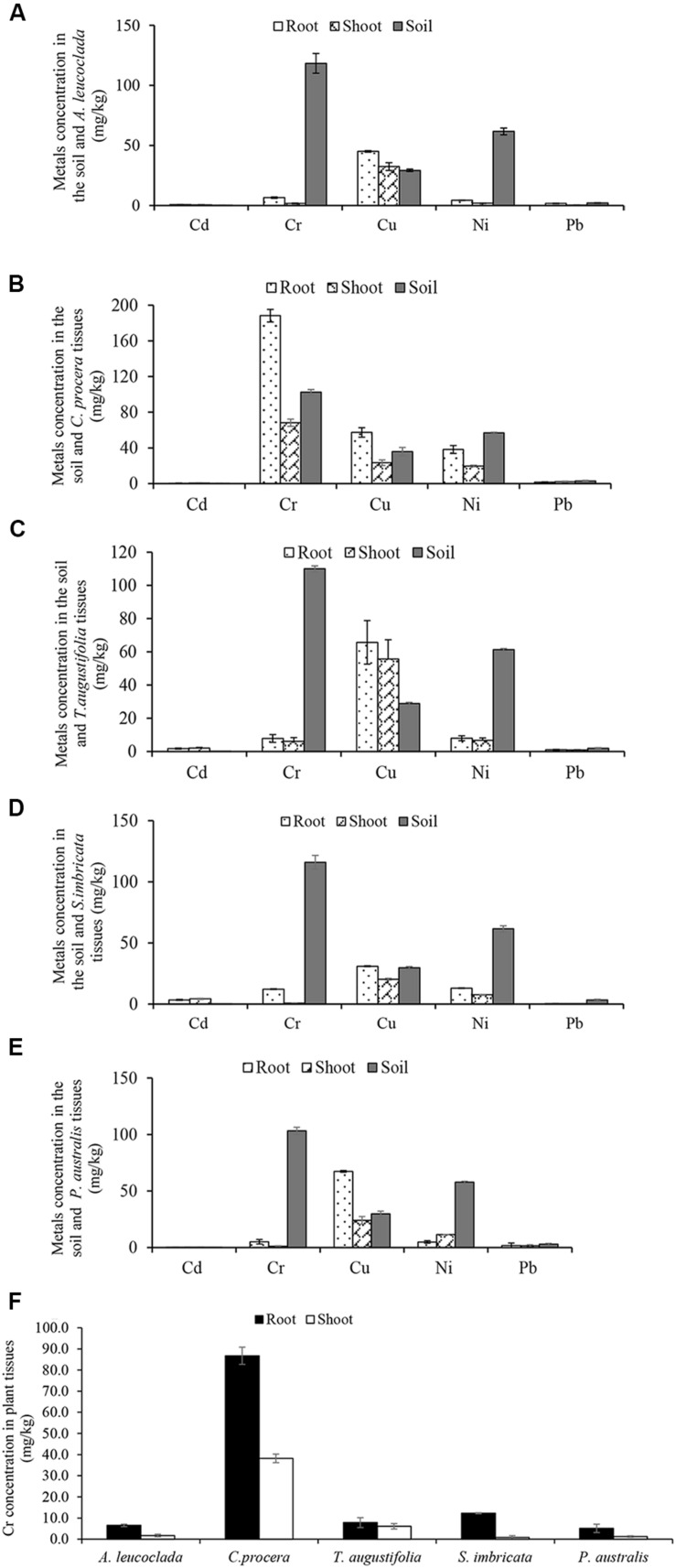
Metals concentration in the soil and plant tissues (Shoot and root) **(A)** *Atriplex leucoclada* **(B)** *Calotropis procera* **(C)** *Typha augustifolia* **(D)** *Salsola imbricata* and **(E)** *Phragmites australis*. Mean concentrations are averages of three replicates (*n* = 3) ± SEM. Mean difference in panel **(F)** (Cr concentration between plant tissues) are statistically significant at *P* < 0.05 level (ANOVA-TUKEY).

Metals concentration in the soil and plant tissues (mg/kg) obtained from the study sites are shown in Figures 1A–D. Soil metals concentration were in the order Cr (111.8) > Ni (60.5) > Cu (29.4) > Pb (2.5) > Cd (0.04). For the plants, accumulation follows the trends; (i) *A. leucoclada* (Figure 1A): Cu (32.4) > Pb (6.7) > Cr (6.5) > Ni (4.3) > Cd (0.7) and Cu (45.0) > Ni (1.8) > Cr (1.7) > Cd (0.6) > Pb (0.3), for root and shoot, respectively. (ii) *C. procera* (Figure 1B): Cr (188.2) > Cu (57.3) > Ni (38.2) > Pb (1.6), Cd (0.4) and Cr (68.2) > Cu (23.4) > Ni (19.7) > Pb (2.0), for root and shoot, respectively. (iii) *T. augustifolia* (Figure 1C): Cu (65.7) > Ni (8.0) > Cr (7.8) > Cd (1.7) > Pb (1.0) and Cu (55.7) > Ni (6.8) > Cr (6.1) > Cd (2.1) > Pb (0.9), for root and shoot, respectively. (iv) *S. imbricata* (Figure 1D): Cu (31.0) > Cr (12.3) > Ni (13.1) > Cd (3.5) > Pb (0.4) and Cu (20.4) > Ni (7.6) > Cd (4.3) > Cr (0.8) > Pb (0.5), for root and shoot, respectively. (v) *P. australis* (Figure 1E): Cu (67.4) > Cr (5.1) > Ni (4.9) > Pb (1.8) > Cd (0.2) and Cu (24.1) > Ni (11.4) > Pb (1.5) > Cr (1.2) > Cr (0.4), for root and shoot, respectively. Soil properties and metals composition are some of the factors that may influence the outcome of correlation analysis (Chen et al., 2014). In this work, the Pearson’s correlation coefficient (*r*) of Cd, Cr, Cu, Ni, and Pb in the soil and the plant tissues (shoot and root) were computed to assess the metals phytoavailability. The results of correlation analysis are shown in Table 2 show both positive and negative correlation between metals concentration in the soil and the plant tissues.

**TABLE 2:** Correlation analysis of metals in the soil and plant tissues (*n* = 3).

**Metals**	***A. leucoclada***		***C. procera***		***T. augustifolia***		***S. imbricata***		***P. australis***	
	**Root**	**Shoot**	**Root**	**Shoot**	**Root**	**Shoot**	**Root**	**Shoot**	**Root**	**Shoot**
Cd	0.88	0.95	0.98	0.99^*^	–0.22	0.74	0.98	0.16	0.46	0.94
Cr	0.41	0.33	0.88	0.99	0.70	0.09	–0.58	–0.92	0.36	–0.25
Cu	–0.82	–0.62	–0.66	–0.41	0.68	0.98	–0.77	–0.68	0.41	0.35
Ni	0.17	0.31	–0.60	–0.83	0.99	0.48	–0.82	–0.94	0.46	–0.95
Pb	0.46	–0.89	−0.99^*^	–0.25	0.99	0.09	–0.58	0.79	0.95	–0.27

*Correlation is significant at 0.05 level.

### Metals Bioconcentration and Translocation

**FIGURE 2:**
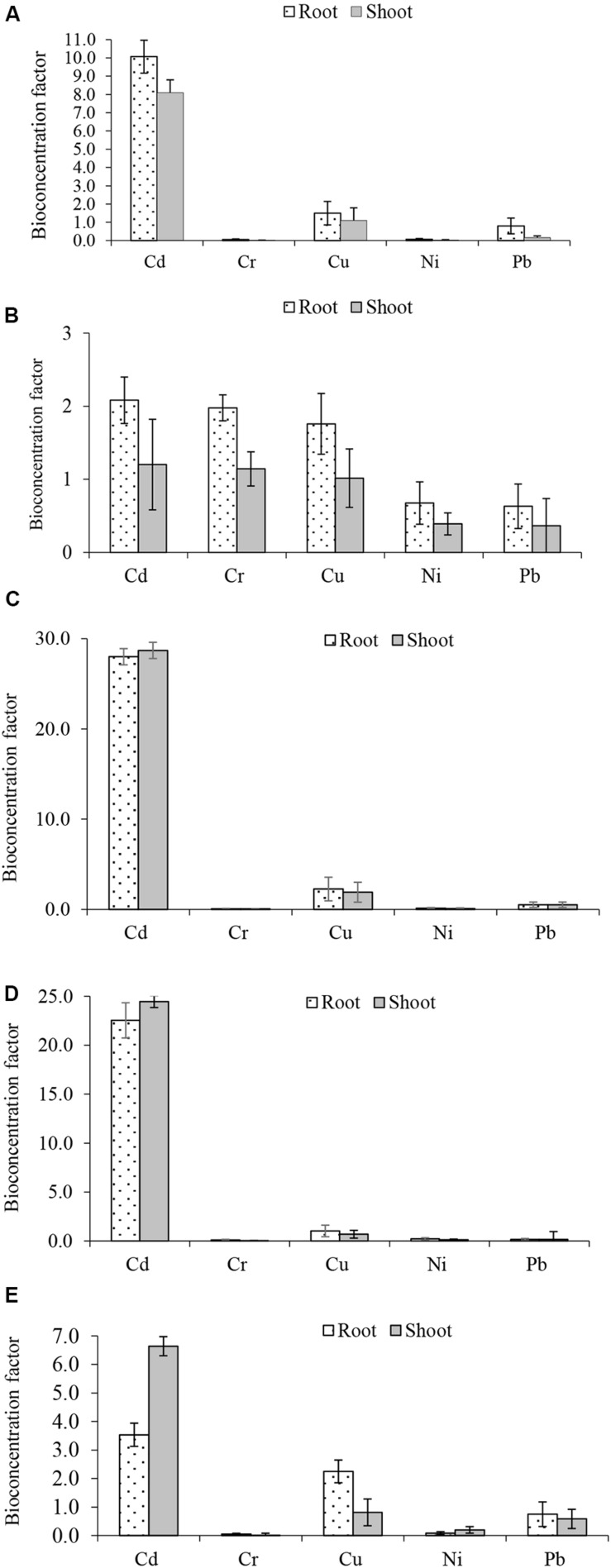
Metals bioconcentration factors **(A)** *Atriplex leucoclada* **(B)** *Calotropis procera* **(C)** *Typha augustifolia* **(D)** *Salsola imbricata* and **(E)** *Phragmites australis*. Mean bioconcentration factors are averages of three replicates (*n* = 3) ± SEM.

**FIGURE 3:**
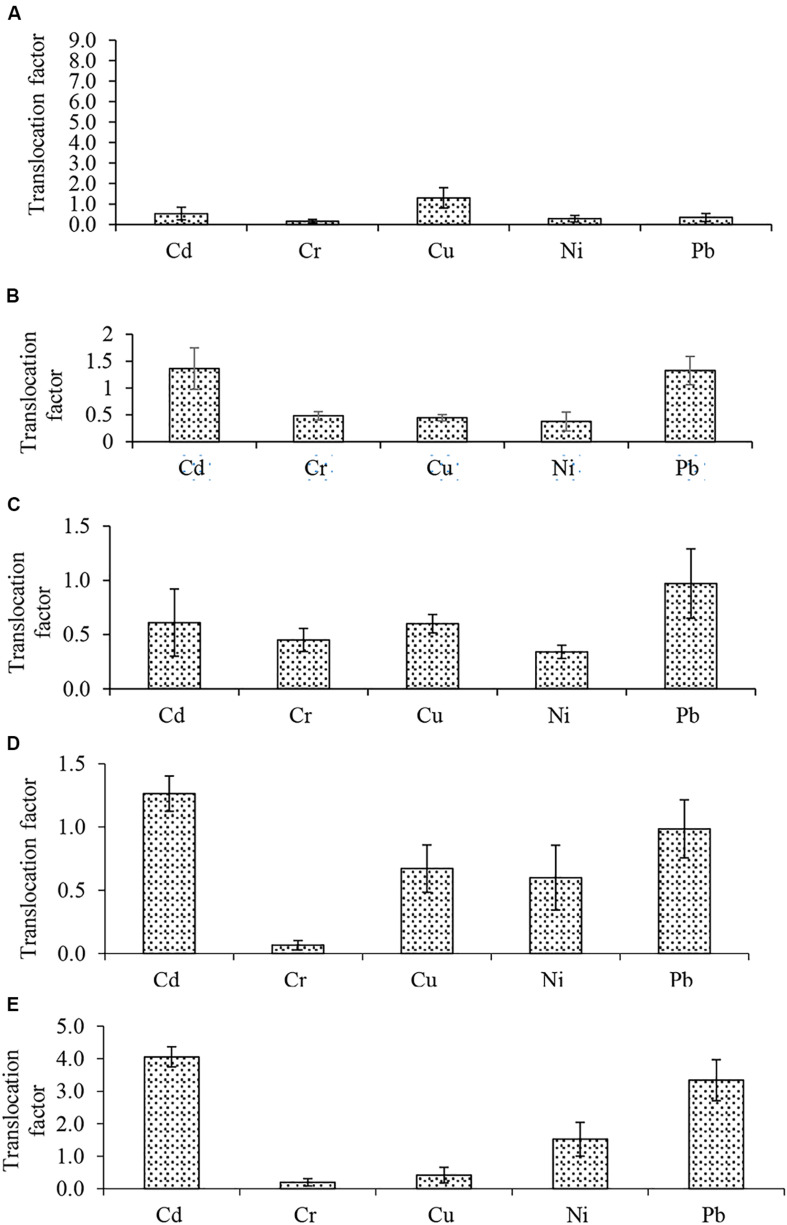
Metals translocation factor **(A)** *Atriplex leucoclada* **(B)** *Calotropis procera* **(C)** *Typha augustifolia* **(D)** *Salsola imbricata* and **(E)** *Phragmites australis*. Mean Translocation factors are averages of three replicates (*n* = 3) ± SEM.

Bio-concentration (BCF) and translocation factor (TF) are important indices in the evaluation of metal bioaccumulation and translocation in plant tissues. BCF estimates metal concentration against concentration in the treatment medium while TF determines whether plants translocate metals to their aerial parts. The BCF and TF of Cd, Cr, Cu, Ni, and Pb in the five plants are shown in Figures 2, 3, respectively. The root BCF for all metals across the five plant species ranges 0–28 (Figure 2), while the shoot BCF for all metals across the five plant species ranges 0–28.7 (Figure 2). Whereas the TF of all metals ranged from 0.1 to 8.3 (Figure 3).

### FTIR Analysis

**FIGURE 4:**
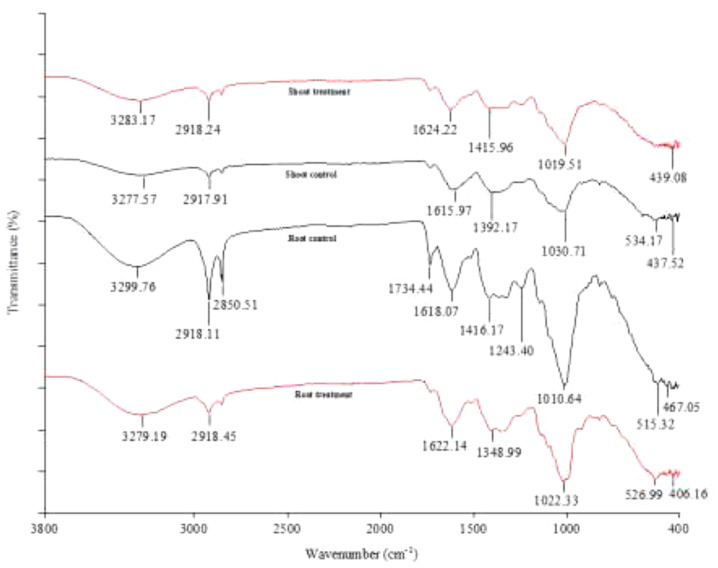
FTIR analysis of *Calotropis procera* treated with 20 mg/L Cr (Treatment) and 0 mg/L Cr (Control).

To understand the binding interaction between *C. procera* tissues and Cr, the root and shoot tissues of *C. procera* grown in greenhouse conditions under 20 mg/kg Cr and the control (0 mg/kg Cr) were compared. The FTIR results of *C. procera* dry biomass with different functional groups (carboxyl, phosphate, amide, and hydroxide) available for metal ions binding is shown in Figure 4. Broad and robust infrared spectra regions between 3500 and 3200 cm^–1^ characterize -OH and -NH stretch. Spectra peaks 3000–2800 cm^–1^ are for –CH_3_ group, 2800–2260 cm^–1^ for – = C-H, 1820–1750 cm^–1^ for – C = O and 1650–1626 cm^–1^ for – C = C (Wen et al., 2018). The regions from 1200–900 cm^–1^ signify C-C, C-O, and C-O-P stretch overlaps (Wolkers et al., 2004) occurring mainly in cellular polysaccharides. Whereas 700−400 cm^–1^ also characterize –OH (Barud et al., 2013). Compared to the control, Cr treated *C. procera* biomass show a higher increase or decrease in peak intensity. In addition, certain peak bands completely disappear in the treatment. The reason can be attributed to an increase in Cr content in Cr treated *C. procera*, which alters infrared spectra absorbance (Arshad et al., 2017; Wen et al., 2018).

### The Antioxidant Enzymes

**FIGURE 5:**
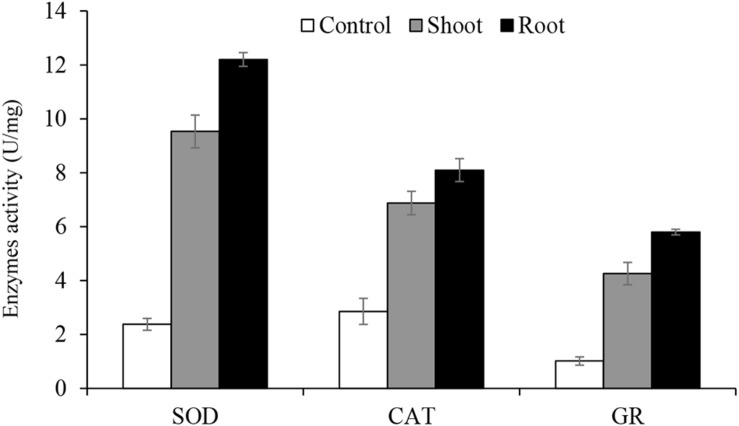
The activities of antioxidant enzymes catalase (CAT), superoxide dismutase (SOD) and glutathione reductase (GR). Means represent averages of three replicates (*n* = 3) ± SEM and are significantly different at *P* < 0.05 level (ANOVA-TUKEY).

The activities of antioxidant enzymes (CAT, SOD, and GR) is presented in Figure 5. Enzymatic assays were performed for *C. procera* grown in greenhouse conditions under 20 mg/kg Cr and the control treatment (0 mg/kg Cr) only. The results show an increase in the activities of all enzymes in Cr treated *C. procera* tissues than the control (Figure 5), indicating that the root suffered from higher Cr stress than the shoot.

## Discussion

In this study, ICP-OES results confirm the presence of the metals Cd, Cr, Cu, Ni, and Pb in different concentrations in the soil and plant tissues collected at Mesaieed industrial area. Previous work by Peng et al. (2016) and Usman et al. (2019) reported the distribution of the same metals on Qatari soil. Overall, ICP-OES data indicates that while Cr was the highest concentrated metal in the soil, all metals, except the barely detectable Cd, bioaccumulates in plants with a preferential concentration in the root. Only Cu recorded higher accumulation in *A. leucoclada* shoot than the root (Figure 1A). A slightly higher shoot Cd concentration was observed in *S. imbricata* (Figure 1D) and *A. leucoclada* (Figure 1A). Our group previously reported the distribution of *Salsosa vermiciluata*, a close relative to *S. imbricata* in the current study at Ras Laffan industrial area of Qatar (Usman et al., 2019). Similarly, consistent with our result, other studies reviewed in Xia et al. (2019) reported Cr accumulation in *T. augustifolia* and *P. australis*.

Previous studies showed that the few plant species *Leersia hexandra, Phragmites communis* (Xia et al., 2019), *Calitriche copacarppa* (Kumar et al., 2014) and *Pennisetum purpereum* (Thayaparan et al., 2015) demonstrated capacity to transfer a considerable amount of Cr into their aerial parts. In this work, *C. procera* stands out with the highest Cr accumulation in both root and shoot tissues compared to Cr concentration in other plant tissues and the soil (Figure 1F). Previous work by Hassan et al. (2015) noted that the perennial shrub plant, *C. procera* (giant milkweed) is a useful bio-indicator of environmental pollution. Furthermore, studies under different conditions showed that the plant is capable of remediating toxic metals including Cd and Pb (D’Souza et al., 2010; Al-Yemni et al., 2011; Lottermoser, 2011; Almehdi et al., 2019). However, except for a closely related species, *C. gigantea* (Sangeetha et al., 2019), to the best of our knowledge, no report on Cr accumulation by *C. procera.*

The correlation analysis reveals a highly significant positive correlation (*r* = 0.99, *p* < 0.05) between Cd concentration in the soil and *C. procera* shoot. While a highly significant negative correlation (*r* = −0.99, *p* < 0.05) between Pb concentration in the soil and *C. procera* root was observed. This indicates air deposition as the most probable source of Pb in the area, due to fossil fuel and leaded gasoline use. Given the low organic matter in the soil, the metal readily bioaccumulates in the plant root. Additionally, Cr concentration in the soil positively correlates with Cr concentration in *C. procera* root (*r* = 0.88, *p* < 0.05) and shoot (*r* = 0.99, *p* < 0.05). Together, the ICP-OES and correlation analysis suggest that *C. procera* easily accumulates Cr in both tissues.

Some of the factors that affect metals bioavailability include plants and metal type, metals form, concentration, and age in the soil, soil pH, and organic matter content (da Conceição Gomes et al., 2016; Shahid et al., 2017; Kumar and Prasad, 2018). Soil pH significantly affects the behavior of Cr by dictating its chemical form (Amin and Kassem, 2012). Generally, metals, including Cr, are more soluble at low or near-neutral pH values. At pH > 8, they tend to precipitates. Of the Cr species, Cr (VI) is generally soluble and more mobile than Cr (III), which ultimately precipitates at a pH > 5.5 (Shahid et al., 2017; Choppala et al., 2018). Cr (VI) is highly toxic with a stable oxidation state. It is associated with oxygen as chromate or dichromate ions. Therefore, the ability of *C. procera* to accumulate high Cr concentration in its tissues can be partly attributed to the neutral pH of the study area (Table 1).

Before compartmentalization, a metal is translocated to the degree that can be described by the TF (Bhatti et al., 2018; Raj et al., 2020a). The evaluation of metals BCF and TF showed the capacity of some of the studied plants to remediate such toxicants. With respect to individual metals, only Cd, Cu and Cr had a BCF of one (1) or more (>1). However, Cu BCF was below one (1) in *S. imbricata* and *P. australis.* For both shoot and root, higher Cd (28.7, 28.0) and Cu (1.5, 1.0) BCF were recorded in *T. augustifolia* (Figure 2C). Cr bioconcentration was reduced in all plants except *C. procera.* All four plants had a BCF less than one (<1), but *C. procera* records a Cr BCF greater than one (>1) at 1.1 and 2.0 for the shoot and root, respectively, (Figure 2B). Additionally, it records BCF values of one or more for Cd and Cu. Further, not only had *C. procera* emerge the only plant with BCF of one or more for Cd, Cu, and Cr, it also accumulates more Ni and Pb compared to other plants. The only exception being Pb in *P. australis* (Figure 2E). Compared to other plants, the BCF suggest that *C. procera* accumulates more metals and capable of freeing soil of Cd, Cu, and Cr. Concerning the TF of all metals, it ranged from 0.1 to 8.3 (Figure 3), indicating differences in the plants’ translocation capacity of the metals. Pb and Cd were the most translocated by all plants. Both metals, except Cd in *T. augustifolia* (Figure 3C), records a TF of one or higher than one in *C. procera* (Figure 3B), *S. imbricata* (Figure 3D) and *P. australis* (Figure 3E). Only *P. australis* demonstrate the capacity to transfer up to three metals (Cd, Ni, and Pb) to the shoot (Figure 3E). Only one plant, *A. leucoclada* (Figure 3A), translocate Cu to the shoot. At 8.3, the TF for Cu was also the highest among all metals. Cr was the least translocated metal with at least three plants recording a TF less than 0.3. Consistent with other similar studies (Kumar and Maiti, 2014; Thakur et al., 2016; Usman et al., 2019), together, the BCF and TF demonstrates that the plant species differ in their metals accumulation and translocation capacity.

Concerning the FTIR results (Figure 4), *C. procera* tissues have different functional groups, including amide, hydroxyl, phosphate, and carboxyl groups. The broad and robust infrared spectra peaks, 3283.17 and 3277.57 cm^–1^ for shoot treatment and control, and 3279.19 and 3299.76 cm^–1^ for root treatment and control, respectively, corresponds to the hydroxyl group functional groups (Wen et al., 2018). Cr has a strong affinity for plant roots due to increased stability when complexed with hydroxyl, carboxylate, and carbonate groups (Usman et al., 2019). A comparison of the root treatment and control indicates the downward shift in peak intensity (3279.19–3299.76 cm^–1^) in the root treatment can be attributed to Cr binding. The drop in peak intensity is due to Cr ions competition for binding to *C. procera* root via the hydroxyl groups. Our result is consistent with the findings of Wen et al. (2018). Wen et al. (2018) studied rhizosphere and non-rhizosphere soil organic matter with and without Cr. Their result showed that the non-Cr treated samples had more hydroxyl (-OH) content compared to the Cr treated samples. About the shoot spectra peaks in the present work (Figure 4), in contrast to the root scenario, a higher band intensity (3283.17 cm^–1^) than the shoot control (3277.57 cm^–1^) was obtained. An explanation could be (i) Cr has a stronger affinity to plant root. Therefore the root FTIR data is the most appropriate to make interpretations of Cr complexation in a 6 weeks experiment. (ii) For metal ions, the hydroxyl functional groups are primarily for essential metal ions binding. However, when transition metals (e.g., Cr) are present, the possibility of substitution exists (Schneider et al., 2001). Therefore, it makes sense to suggest that the drop in peak intensity is due to the presence of more essential metals (e.g., Na^+^, Ca^2+^, H^+^, Mg^2+^, K^+,^ and Fe^2+^) in the shoot control than the shoot treatment. The peaks denote alkanes and carboxylic groups present in the samples at 2918.24 and 2917.91 cm^–1^ for shoot treatment and control, and 2918.45 and 2918.11 cm^–1^ for root treatment and control, respectively. The peaks at 1624.22 and 1615.97 cm^–1^ for shoot treatment and control, and 1622.14 and 1618.07 cm^–1^ for root treatment and control, respectively, denote amines, aromatics, and alkenes presence. Previous studies by Siva and Udayakumar (2015) and Sangeetha et al. (2019) reported the involvement of amino groups in Cr uptake in *Allium cepa* and *C. gigantea*, respectively. Both samples spectra peaks at 1415.96 and 1392.17 cm^–1^ for shoot treatment and control, and 1348.99 and 1416.17 cm^–1^ for root treatment and control, respectively, show the presence of C-C and C-H groups. The regions from 1200−900 cm^–1^ are for C-C, C-O, and C-O-P stretches (Wolkers et al., 2004) occurring mainly in cellular polysaccharides. Whereas 700−400 cm^–1^ also characterize –OH (Barud et al., 2013).

Further, the FTIR data shows that Cr induced the most changes in the organic constituent of *C. procera* root than the shoot (Figure 4). The root control bears ten (10) spectra peaks compared to seven (7) in the root treatment. In the shoot, control showed seven (7) peaks compared to six (6) in the treatment. For instance, in the root treatment, no peaks were corresponding to the bands at 2850.51, 1734.44, and 1243.40 cm^–1^ in the shoot control (Figure 4). Additionally, for the most peaks, the shift in bands intensity between treatment and control was higher in the root compared to the shoot. This observation is consistent with the reports of two previous studies involving a closely related species, *C. gigantea* (Ramamurthy and Kannan, 2007; Sangeetha et al., 2019), where only minor changes in peak values were observed in Cr polluted leaves. Based on leaves FTIR data, Sangeetha et al. (2019) concluded that *C. gigantea* tolerates Cr by exclusion mechanism. However, it is essential to note that our work differs from that of the previous authors in that FTIR results are exclusively from *C. procera* tissues (root and shoot) following Cr treatment under greenhouse conditions. Therefore, based on ICP-OES, BCF and TF values from field samples, and FTIR results (from the greenhouse experiment), we conclude that the mechanism of Cr tolerance in *C. procera* involves complexation, uptake, and translocation. The Cr ions mainly bind to the root via hydroxyl and carboxylic groups (Zhang et al., 2008; Wen et al., 2018), and transfer to other plant parts. Our conclusion is consistent with the findings of authors in studies involving plant root and soil organic matter (Zhang et al., 2008; Wen et al., 2018).

In this work, the result of SOD, CAT, and GR assay show increased activity of all three enzymes in *C. procera* tissues due to Cr stress (Figure 5). Shahid et al. (2017) and Xia et al. (2019) reviewed several studies supporting our result in various plants, including model species, *Arabidopsis thaliana*, and *Oryza sativa.* Higher enzymatic activities in the root supports our FTIR results (Figure 4), where apparent changes in organic constituents and shift in peaks intensity were observed in the root due to Cr complexation than the shoot. Furthermore, for both shoot and root, SOD activity was the highest (up to 12.2 U/mg), particularly in the root, suggesting its critical role in *C. procera* antioxidative system. CAT is next, while GR demonstrates the least activity. Such differences can be attributed to their specific roles in ameliorating Cr stress in *C. procera*. Many studies reported an increase in the activities of SOD due to Cr treatment in plants; examples are in *Pisum sativum* (Tripathi et al., 2015) and *Corchorus olitorius* (Islamit et al., 2014). Similarly, our result is consistent with the report of Rivas et al. (2017), where SOD was activated following *C. procera* exposure to drought stress. Previous studies by Daud et al. (2014) and Islamit et al. (2014) reported increased activities of CAT in *Gossypium hirsutum* and *Corchorus olitorius*, respectively. Further, enhanced GR activity was reported in *Corchorus olitorius* (Islamit et al., 2014). However, in a separate study, Kováčik et al. (2014) noted an increase and decrease in GR activities in *Hordeum vulgare* and *Matricacaria chamomilla*, respectively. According to Maiti et al. (2012), changes in the enzymatic activities account for the elimination of ROS and improvement of stress conditions in *C. procera.* Therefore, the increase in the activities of SOD, CAT, and GR in 20 mg/kg Cr treated *C. procera* indicates that the enzymes play protective role in the plant response mechanism to Cr stress.

It is worth noting that the results presented in this work are limited to deciphering Cr tolerance and uptake mechanism from the metal translocation and plant antioxidative system perspective. However, we recognize that proteins regulates ROS signaling and the expression of such proteins changes due to metal stress, including Cr. In fact, increased protein synthesis due to metal stress in plants is one of the major cellular metabolic processes. The mitogen-activated protein (MAP) kinase pathways regulate such processes, which serves as signaling system against oxidative stress (Shahid et al., 2017; Kumar and Prasad, 2018). The signaling occurs through multiple stages of reaction, which modify gene expression and ultimately protein synthesis (Sidhu et al., 2016). Therefore, our future work will focus on the differential expression of proteins, particularly those that relates to stress response, such as the heat shock proteins family due to Cr exposure in *C. procera*.

## Conclusion

In the present study, results confirmed the occurrence of Cd, Cr, Cu, Ni, and Pb in the soil at Mesaieed industrial area. Of such metals, Cr concentration was the highest, while Cd recorded the least. Due to high accumulation in the root, *C. procera* is capable of phyto-stabilizing the metal. It proves, therefore, a potential candidate for Cr phytoremediation, particularly in arid and semi-arid soil. Binding interaction between the metal and *C. procera* root is by ionic exchange via the hydroxyl and carboxylic groups. The plant’s Cr tolerance and bioaccumulation involve complexation, uptake, and translocation. The increased activities of antioxidant enzymes were critical to maintaining cellular homeostasis via redox signaling in the arid plant. Therefore, it can be concluded that *C. procera* partly dealt with Cr toxicity by reducing free radicals via its antioxidant system. However, to unravel the complete mechanisms governing Cr detoxification following uptake and translocation in *C. procera* tissues, it is also essential to study the regulation at a molecular level.

## Data Availability Statement

All datasets presented in this study are included in the article/supplementary material.

## Author Contributions

KU, MA, and MA-D conceived the idea. KU, MA, HA, and MA-D designed the experiments and interpreted, and analyzed the datasets. KU wrote the manuscript. KU, MA, HA, and MA-D revised the manuscript. All authors contributed to the article and approved the submitted version.

## Conflict of Interest

The authors declare that the research was conducted in the absence of any commercial or financial relationships that could be construed as a potential conflict of interest.
